# Molecular analysis of 18S rRNA gene of *Cryptosporidium* parasites from patients living in Iran, Malawi, Nigeria and Vietnam

**Published:** 2012

**Authors:** Salman Ghaffari, Narges Kalantari

**Affiliations:** 1 *Parasitology and Mycology Deparment, Faculty of Medicine, Babol University of Medical Sciences, Babol, Iran.*; 2 *Cellular and Molecular Biology Research Center (CMBRC), Babol University of Medical Sciences, Babol, Iran.*

**Keywords:** 18S rRNA, *Cryptosporidium*, diarrhea, developing countries

## Abstract

*Cryptosporidium *species are one of the most common causes of gastrointestinal infection in humans around the world. This study has aimed to investigate the hyper variable region of the 18S rRNA gene in *Cryptosporidium* for exact parasite identification.

DNA was extracted from 26 fecal samples from which initially *Cryptosporidium *oocysts were identified by Ziehl-Neelsen acid-fast , Auramine phenol and ELISA techniques. Nested PCR, targeting the most polymorphic region of the 18S rRNA gene and genotyping was performed by restriction endonuclease digestion of the PCR product followed by nucleotide sequencing and phylogenic analysis.

Among 26 isolates analyzed, three species of *Cryptosporidium *were identified; 38.5% of the isolates were *C. hominis* while 53.8% of the isolates were *C. parvum* and 7.7% of the isolates were *C. meleagridis*, which the last two species have the potentially zoonotic transmission. The only 11T subtype of *C. hominis* was demonstrated. These strains clustered distinctly into either human or animal origin regardless of the geographical origin, age, or immunity status of the patients.

In summary, this work is the first report of *C. meleagridis *infecting human in Iran. Moreover, it suggested that multi-locus study of *Cryptosporidium* species in developing countries would be necessary to determine the extent of transmission of cryptosporidiosis in the populations.

*Cryptosporidium *species are frequent agents of gastrointestinal infection in humans, domestic animals, and other vertebrates through the world (1). The diagnosis of cryptosporidiosis is usually made by using direct detection by microscopy stained tissues or fecal material, enzyme immunoassays for antigen detection and molecular techniques. Microscopy and staining methods have suffered from the problem of distinguishing both *Cryptosporidium* from different species of this parasite and from other fecal components of similar size and shape such as yeasts and fungus, thus affecting the sensitivity and specificity (2).

Various species of these parasites can be distinguished only by using molecular techniques, even though a number of important biological differences have been also recognized (3). Application of molecular studies in the classification of *Cryptosporidium *species has led to increased identification of the diversity of the species infecting humans (4). Until now, at least twenty-two species are accepted as valid in the *Cryptosporidium *genus based on polymerase chain reaction (PCR) assays. The molecular studies have reported that more frequent *Cryptosporidium* species in humans are C*. parvum *, *C.hominis *and *C. meleagridis* (1, 5-6).

Human cryptosporidiosis is generally transmitted through two routes. One route occurs from human-to-human (anthroponotic transmission) for C. hominis and C. parvum and another occurs from animal-to-human (zoonotic transmission) for C. parvum (7). In developed countries, epidemic cryptosporidiosis can occur in children and adults as food-borne or waterborne outbreaks (8). In developing countries, Cryptosporidium infections occur mostly in children younger than 5 years, with peak occurrence of infections and diarrhea in children under 2 years old (9-10).

There have been few reports on the molecular analysis of the various *Cryptosporidium* species in diarrheic or non diarrheic individuals in Iran. Also, there are limited or no molecular studies of cryptosporidiosis in many other developing countries such as Malawi, Nigeria, and Vietnam. The current study has investigated the hypervariable region of the 18S rRNA gene in *Cryptosporidium* for exact identification of parasites isolated from Iran, Malawi, Nigeria and Vietnam. It also evaluated the extent of variation in sequences from the isolates within each species and their effect on the application of this gene target to a phylogenetic analysis of *Cryptosporidium*.

## Materials and Methods

**Sample collection and DNA extraction.**

*Cryptosporidium *oocysts were recovered from human fecal samples collected from Iran (21 samples), Malawi (2 samples), Nigeria (1 sample) and Vietnam (2 samples). These samples were frozen at -80°C or stored at 4ºC either without preservative or with 75% ethanol (11), or 2.5% potassium dichromate or 10% formalin. 

Specimens stored in potassium dichromate and formalin were washed off thoroughly by centrifugation and rinsing with cold distilled water six times in order to remove preservative prior to DNA extraction. A pea sizes of sample (about 200 mg), the frozen fecal sample or approximately a 100 µl final suspension of each sample in potassium dichromate and formalin was suspended in 500 µl of ASL buffer and then vortexed for 30 seconds. Oocysts were ruptured by subjecting them to a freeze-thaw cycle of +80ºC for 15 min and–80ºC for 30 min. DNA was extracted from fecal samples by the QIAamp® DNA stool mini kit (QIAGEN Ltd., Crawley, West Sussex, UK) according to the manufacturer's instructions. The DNA was further purified following the manufacturer-suggested procedures included in the QIAamp DNA Stool Mini Kit and stored at -20°C until it was used for PCR assays. 

**Nested PCR amplification and RFLP analysi**s

The 18S rRNA gene was amplified using the nested PCR protocol as described by Xiao *et al*., 1999 (12). A primary PCR step gave a PCR product that was about 1,325 bp long, was followed by a secondary amplification of an internal fragment with a length of approximately 840 bp. This fragment has been shown to be very specific to *Cryptosporidium* species and for genotyping. Then, the different species were identified by applying restriction digestion of the secondary product.

The primary PCR was amplified by using primers 5′-TTC TAG AGC TAA TAC ATG CG-3′ and 5′-CCC TAA TCC TTC GAA ACA GGA-3′. Each reaction mixture contained 10 μl of Perkin-Elmer 10× PCR buffer, 12 μl MgCl_2_, 8 μl dNTPs, 2.5 μl of each primer, 0.5 μl of *Taq* DNA polymerase, 2 μl of DNA template, 1 μl bovine serum albumin (1%) and HPLC water to make a final volume of 50 μl. The PCR reactions were carried out in a Techne Thermal cycler (Techne Ltd., Cambridge, UK) using the following PCR protocol; an initial hot start at 94°C for 3 minutes, followed by 35 cycles, each consisting of 94°C for 45 seconds, 55°C for 45 seconds, and 72°C for 1 minute; and a final extension step at 72°C for 7 minutes.

Positive and negative controls were included in every PCR reaction. PCR products were visualized by UV after electrophoresis in 1% agarose gel and staining with ethidium bromide. For the secondary PCR step, a PCR product that was 819 to 825 bp long (depending on the species) was amplified by using 2 μl of the primary PCR product and primers 5′-GGA AGG GTT GTA TTT ATT AGA TAA AG-3′ and 5′-AAG GAG TAA GGA ACA ACC TCC A-3′. The PCR mixture and cycling conditions were identical to the conditions used for the primary PCR step, except that 6 μl of MgCl_2_ (3 mM) was used in the PCR mixture (12).

RFLP analysis of the secondary PCR product was carried out by digesting with SspI for species identification and with *Vsp*I for genotyping of *Cryptosporidium* species as described previously (12). Briefly, for the restriction digestion (37°C for 80 minutes), each reaction mixture contained 15 µl of the secondary product, 1 µl of *Ssp*I (20 U), 2.5 µl of restriction buffer and 11.5 µl of HPLC water to make a final volume of 30 μl for species identification. Furthermore, *Vsp*I (Boehringer Mannheim, Germany) at the same concentration described for SspI was used for genotyping. The digestion products were separated on a 2% agarose gel and visualized after ethidium bromide staining. Isolates were assembled according to their RFLP patterns, and a representative of each group was selected for sequence analysis.

**Sequencing study**

PCR products were sequenced directly following purification using either QIAquick Gel Extraction Kit (QIAGEN, West Sussex, UK) or the MicroSpin Columns Kit (Amersham Biosciences, Buckinghamshire, UK).

The ChromasPro programme version1.3 (2003, Technelysium Pty. Ltd., Australia) was used to read all the amplicon sequences. DNASTAR version 5.06 (2003) was used for editing the consensus sequences and multiple alignments of the DNA sequences. To confirm the identity of the sequences from the GenBank, Blast Local Alignment Search Tool (BLAST) searches in National Center for Biotechnology Information (NCBI) were undertaken. The DNA distance based Neighbor Joining (NJ) analysis was performed by using the phylogenetic analysis software TREECON for windows version1. 3b. The GC/AT content was determined by using a programme on the European Bioinformatics Institute (EBI) site (www.ebi.ac.uk). 

**Nucleotide sequence accession numbers.**

The five sequences used in this study have been deposited in the GenBank database under the accession no DQ002918.1, DQ010950.1, DQ010951.1, JX547008.1 and JX547010.1.

## Results

**Fig 1:**
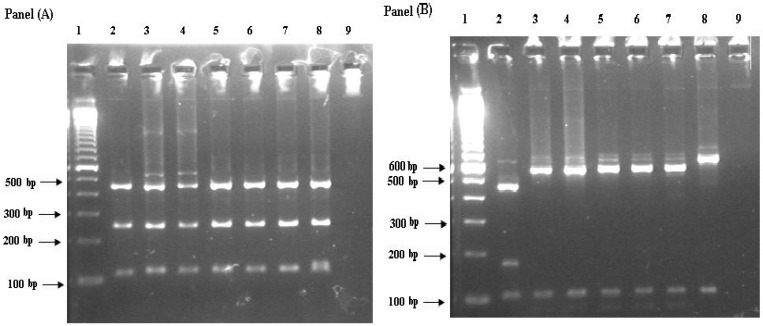
The RFLP for 18S rRNA gene. Panel (A) showing the results of restriction digestion with *Ssp*I. Panel (B) shows the digestion patterns with *Vsp*I, showing bands expected for *C. meleagridis *(lane2), *C. hominis *(lanes3-7), *C. parvum* and positive control (lane 8) and negative control (line 9). Lane 1 is marker (1000 bp).

The nested PCR reaction of 18S rRNA gene, using specific primers, demonstrated an expected band on agarose gel in 13 out of 26 (50%) samples. The secondary PCR yielded amplicons of sizes between 833 to 837 bp depending on the *Cryptosporidium* species. RFLP analysis of the nested-PCR products showed that 7 (53.8%) samples were *C. parvum *and that 5 (38.5%) were *C. hominis*. Single isolate of *C. meleagridis *(7.7%) was also recognized (Figure 1 ). All *C. parvum*, 2 *C. hominis *and 1* C. meleagridis *isolates were from Iran. The three *C. hominis *isolates were from Malawi, Nigeria and Vietnam. The distribution of *Cryptosporidium *species by patients' origin and immunity status is presented in Table 1.

**Table 1:** The distribution of *Cryptosporidium* species by patients' origin and immunity status

**Source**	**Number**	**Patients**	**Preservation**	**Status**	**Species**
*(a) Samples from Iran*					
Mazandaran	3	Children	Cool (4ºC)/75% Ethanol	Diarrheic	*C. meleagridis* (1 isolate)
Tehran	1	Child	2.5% K..dichromate^1^	ALL^2^	*C. hominis*
Tehran	1	Adult	2.5% K.dichromate	HIV^+^	-
Tehran	1	Adult	10% Formalin	HIV^+^	-
Shahrekord	15	Children	75% Ethanol	Diarrheic	*C. parvum* (7 isolates) + *C. hominis* (1 isolate)
*(b) Samples from other regions*					
Malawi	2	Children	Frozen (-80ºC)	Diarrheic	*C. hominis*(1 isolate)
Nigeria	1	Children	Cool (4 ºC)	Diarrheic	*C. hominis*
Vietnam	2	Children	Frozen(-80ºC)	Diarrheic	*C. hominis*(1 isolate)

^1^K.dichromate: Potassium dichromate

^2^ALL: Acute Lymphoid Leukaemia

**18S rRNA gene sequence analysis**

**Fig 2:**
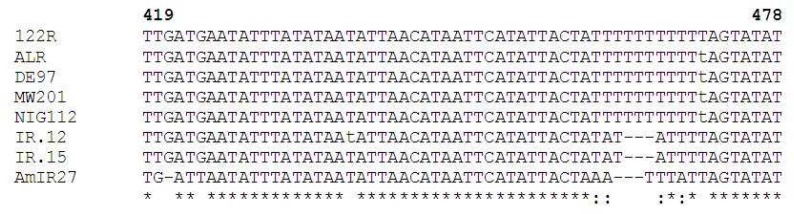
Nucleotide positions in the aligned sequences of *Cryptosporidium* species of the 18S rRNA gene. * denotes nucleotides identical to those of the 122R (*C. hominis*) sequence,: indicate differences of nucleotide of *C. meleagridis* with *C. parvum* and *C. hominis*, - indicate the absence of sequence information for a particular isolate

Partial sequences from the 18S rRNA gene were obtained from *C*. *hominis *(5 isolates), *C. parvum* (7 isolates) and *C. meleagridis* (one isolate). Depending on the species, the lengths of the gene fragments varied from 789 to 837 bp in *C. hominis* and all of the *C. parvum* isolates had the shortest length. The 18S rRNA gene of *Cryptosporidium* species were AT rich with A and T % content of 64.58% in *C. meleagridis*, 64.36% in *C. parvum *and 64.42% in *C. hominis* isolates. Based on the species, there is a region of high AT content about 300-400 nucleotides into the sequence. The characteristic AT rich region seen in both *C.* *hominis* and *C. parvum* and the specific T-repeat at position approximately 460-470 seen in *C. hominis* affected the sequencing efficiency and caused difficulty with assembling the sequence with the software (Fig. 2). Therefore, manual assembling of the sequences was done for this collection. 

A BLAST search (NCBI) of the sequences confirmed their identity which was in agreement with the results for the majority of the species identifications obtained by PCR-RFLP analysis of all isolates of *Cryptosporidium* species. Each of the* C. parvum* isolates from Iran showed 99-100% identity to the published isolate HMa (Accession number AY204238). One *C. meleagridis* isolate from Iran showed 99% sequence identity with the published strain TU1867 (Accession number AY166839). Both isolates of *C. hominis* from Iran showed 100% similarity to the published strains of *C. hominis* of UG502 and SI19 (Accession numbers AF481962 and AJ849464), respectively. The isolates from Vietnam and Nigeria had 100% and from Malawi 99% sequence identity to the published strain of UG502 *C. hominis* (Accession number AF481962). 

**Fig 3:**
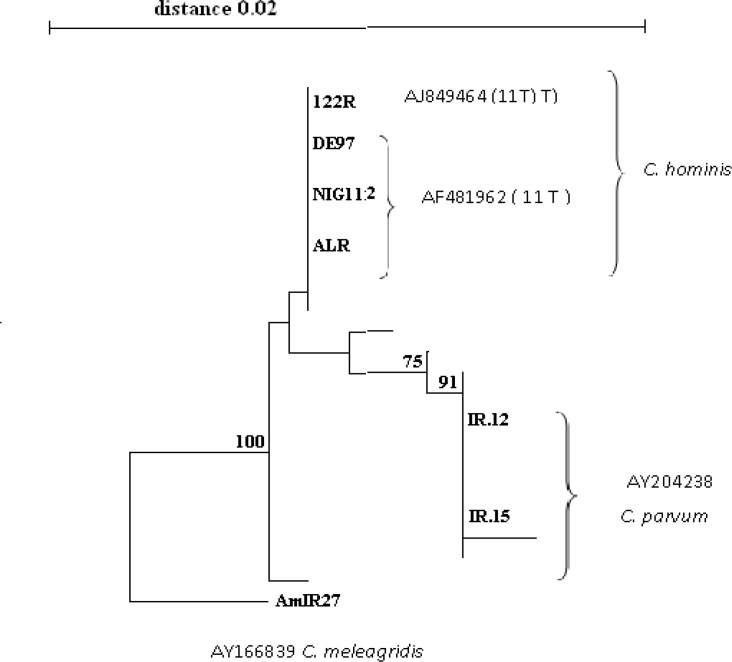
Phylogeny of *Cryptosporidium* isolates by a rooted NJ-tree based on 18Sr RNA. The numbers on branches are bootstrap values greater than 70%.The scale bar indicates an evolutionary distance of 0.02 nucleotides per position in the sequence. The reference sequences accession numbers are inserted. The number of T in T-repeat region in *C. hominis* isolates are shown in parentheses. The cods are as follow: ALR: Tehran, AmIR: Amol; 122R, IR12 and IR15: Shahrekord, Iran; MW: Malawi, NIG: Nigeria, DE: Vietnam

Phylogenetic relationships of the isolates from the different geographical regions were evaluated by analysis using the neighbor - joining phylogram method (Fig. 3). The findings demonstrated three different *Cryptosporidium* groups including *C. hominis, C. parvum* and *C.meleagridis*. *C.meleagridis* was used as the out-group as this species showed least similarity to the others. In the NJ method the three *Cryptosporidium* species examined in this study formed two clades with the full statistical reliability (100%). One clade contained C*. meleagridis* at the out-group; the other clade consisted of *C. hominis* and *C.* *parvum.* At approximately position 461 on the 18S rRNA gene the *C. parvum* isolates displayed the sequence TATATTTT whereas *C. hominis* isolates exhibited the sequence TTTTTTTTTTT. This difference together with other small differences in this region appears to be responsible for the topology of the tree. The cases of *C. hominis* have a sequence of 11 T (Fig. 2).

Among 8 isolates sequences of 18S rRNA gene in this study, one isolate was excluded from NJ phylogram due to the presence of a shorter sequence. This isolate was from Malawi (MW201, *C. hominis*). 

## Discussion

In the current study, we identified similarities and differences in infecting species among cases from four different geographical countries. Furthermore, these findings demonstrated that *C. parvum* was more frequently detected than *C. hominis*. These results are in agreement with results obtained from other studies indicating that *C. parvum* was the most prevalent species found in humans (13-16). Three species including *C. parvum *(70%), *C. hominis* (20%) and *C. meleagridis* (10%) were identified in isolates from Iran which exhibited considerable diversity. Presence of at least three species in the sample collection from Iran indicates that different transmission cycles of cryptosporidiosis may occur in this country. For example, in the case of *C. meleagridis *in this study, the patient was a diarrheic two years old girl from an urban area of Amol city, Mazandaran Province, Northern Iran; who was referred to a private diagnostic laboratory. In spite of many attempts, there was no more information available such as her immunity, contact with animals, etc... This work is also the first report of *C. meleagridis *infecting human in Iran. Human infection with several subtypes of this parasite (17) may relate to contact with aviary birds (18) a history of foreign travel and human immunodeficiency virus-positivity (19). *C. meleagridis* may be a common pathogen to a number of hosts and indeed may have originally been a parasite of mammals that has subsequently become established in birds (20). 

*C. hominis* was the only specie identified in isolates from Malawi, Nigeria and Vietnam. This result is similar with the finding of Gatei *et al*. (2003) from Vietnam, but is different from their isolates from Malawi which were *C. hominis* and *C. parvum *(13). *Recent studies showed that* *C. hominis* *is the predominant specie in Nigeria* (21-22) and Malawi (23). These findings indicate that the pattern of *Cryptosporidium* distribution in Iran is different from those in other countries included in the current study.

On the other hand, of 26 positive samples of *Cryptosporidium* oocysts which were obtained by screening methods, only 13 (50%) samples yielded a PCR product for the 18S rRNA gene. Failure to yield PCR products of samples could be related to various factors and has some explanations. These are described as below as: the relatively low oocysts count in some of the samples (24); the presence of PCR inhibitors in fecal samples such as bile acids, hemoglobin and complex polysac-charides even though they are present at low concentration (25); extraction procedures and failure to remove sample fixatives and preservatives and oocysts age (26), failure of cell lysis, nucleic acid degradation and capture or of an insufficient amount of DNA (27).

However, there are several genotypic markers to differentiate some species and subspecies within the genus *Cryptosporidium*. Nested-PCR/RFLP and sequencing for 18s rRNA gene have been reported to be appropriate to differentiate the parasite species (28).

Furthermore, previous studies have shown an extensive application of this gene in the phylogeny of* Cryptosporidium*, with the indication that the presence of heterogeneous copies within the genome of a single organism does not affect the overall phylogenetic position of the organism. 

The examination of the 18S rRNA gene sequence by determining the rolling average of 100 bases over the length of the gene showed a region where the AT content was 90%. The sequences of the section containing the 11T repeats in *C. hominis* isolates were aligned and all showed the same repeat number at this region. (Fig2). However, a study showed that isolates of *C. hominis* had 11, 10, 8 or 6 T in their sequence (29). 

Although, the current study successfully identified three different *Cryptosporidium* species by analyzing 18S rRNA gene, but there are some doubts about the appropriate resolution of this gene. Our results showed that cautiousness in the application of this loci fragment in fine scale phylogeny is necessary. However, this gene target remains an important tool for the precise identification of *Cryptosporidium *species especially due to the wide application of a single set of primers for the different species. Another limitation of this work was the samples size and the lack of sufficient epidemiological data to elucidate the source of parasites and reasons responsible for the epidemiology of cryptosporidiosis.

In conclusion, this work is the first report of *C. meleagridis *infecting human in Iran. Moreover, these results showed the limitations of the use of this gene and indicate the

need for further research particularly on adequate samples size and application of a multi-locus study of *Cryptosporidium* species in developing countries to determine the extent of transmission of cryptosporidiosis in the populations. 
